# Two cases of atrial myxoma with calcification and ossification as the main features

**DOI:** 10.1186/s13019-024-02876-8

**Published:** 2024-06-26

**Authors:** Yafei Yin, Juan Deng, Yuan Liu, Jingxin Zheng, Yun Zhang, Qizhi Bai, Yali Xu, Guoliang Yang

**Affiliations:** 1 Department of Ultrasound, The Second Affiliated Hospital of Army Medical University (Third Military Medical University), Chongqing, 400037 China; 2 Department of Pathology, The Second Affiliated Hospital of Army Medical University (Third Military Medical University), Chongqing, 400037 China; 3 Department of Cardic surgery, The Second Affiliated Hospital of Army Medical University (Third Military Medical University), Chongqing, 400037 China; 4 Department of Radiology, The Second Affiliated Hospital of Army Medical University (Third Military Medical University), Chongqing, 400037 China

**Keywords:** Cardiac myxoma, Calcification, Ossification, Echocardiography, Transesophageal echocardiography, Contrast transthoracic echocardiography

## Abstract

**Background:**

Cardiac myxomas are the most common type of primary cardiac tumors in adults, but they can have variable features that make them difficult to diagnose. We report two cases of atrial myxoma with calcification or ossification, which are rare pathological subgroups of myxoma.

**Case presentation:**

A 47-year-old woman and a 35-year-old man presented to our hospital with different symptoms. Both patients had a history of chronic diseases. Transthoracic and transesophageal echocardiography revealed a mass in the left or right atrium, respectively, with strong echogenicity and echogenic shadows. The masses were suspected to be malignant tumors with calcification or ossification. Contrast transthoracic echocardiography(cTEE) showed low blood supply within the lesions. The patients underwent surgical resection of the atrial mass, and the pathology confirmed myxoma with partial ossification or massive calcification.

**Conclusion:**

We report two rare cases of atrial myxoma with calcification or ossification and analyze their ultrasonographic features. Transthoracic echocardiography and cTEE can provide valuable information for the diagnosis and management of such mass. However, distinguishing calcification and ossification in myxoma from calcification in malignant tumors is challenging. More studies are needed to understand the pathogenesis and imaging characteristics of these myxoma variants.

**Supplementary Information:**

The online version contains supplementary material available at 10.1186/s13019-024-02876-8.

## Background

Cardiac myxomas (CM) are the most common type of primary cardiac tumors in adults [1], and the left atrium is the most frequently involved site, with 75% of the CM originating from the interatrial septum (IAS). CM that may cause serious complications, such as embolism, valve obstruction, and heart failure, have to be diagnosed and treated promptly. This is crucial for improving the prognosis and quality of life of patients [2, 3]. CM have variable features in terms of location, shape, composition, and vascularization, which can make them difficult to distinguish from other lesions. Moreover, cardiac myxomas, in rare instances, can undergo calcification or ossification. This can be misleading on echocardiography, as it may mimic the imaging characteristics of malignant tumors [3]. In this report, we present the two cases of CM patients who had calcification or ossification in their tumors and discuss the distinction between the two in echocardiography.

## Case 1

**Fig. 1:**
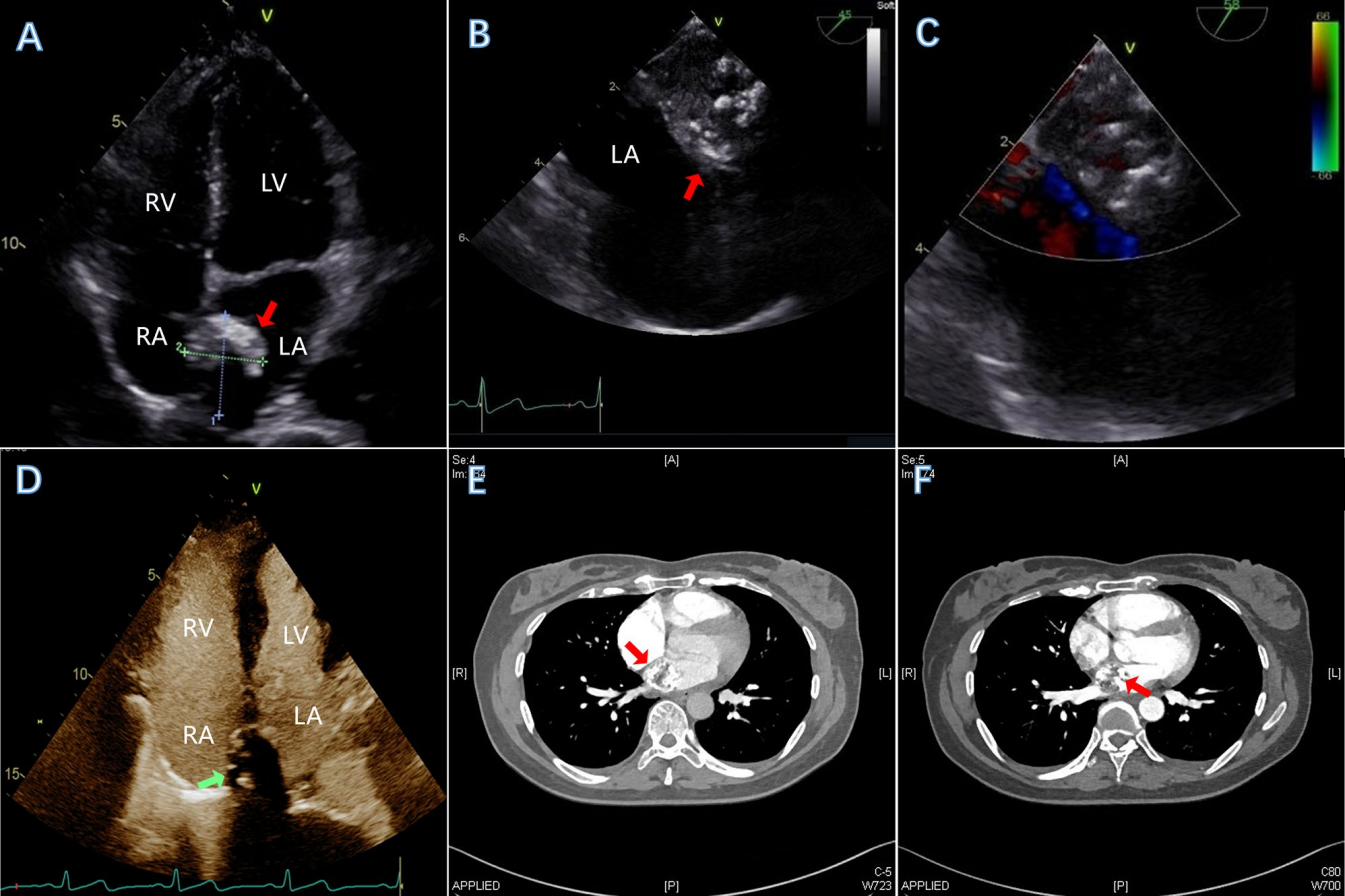
Multiparametric imaging. In the four-chamber view on TTE, a mass was detected in the left atrium, adjacent to the IAS. The mass was hyperechoic with significant posterior acoustic shadowing and exhibited minimal mobility, as indicated by the red arrow in image (**A**). TEE (two-dimensional and color Doppler ultrasound) showed left atrial mass(**B** and **C**). The cTTE revealed a small amount of contrast agent (green arrow) infiltrating the mass(**D**). CTA demonstrated that the mass in the left atrium was heterogeneously contrast enhanced during the venous phase and showed further enhancement during the arterial phase (**E**-**F**). *Note* LA, left atrium; LV, left ventricle; RA, right atrium; RV, right ventricle

**Fig. 2:**
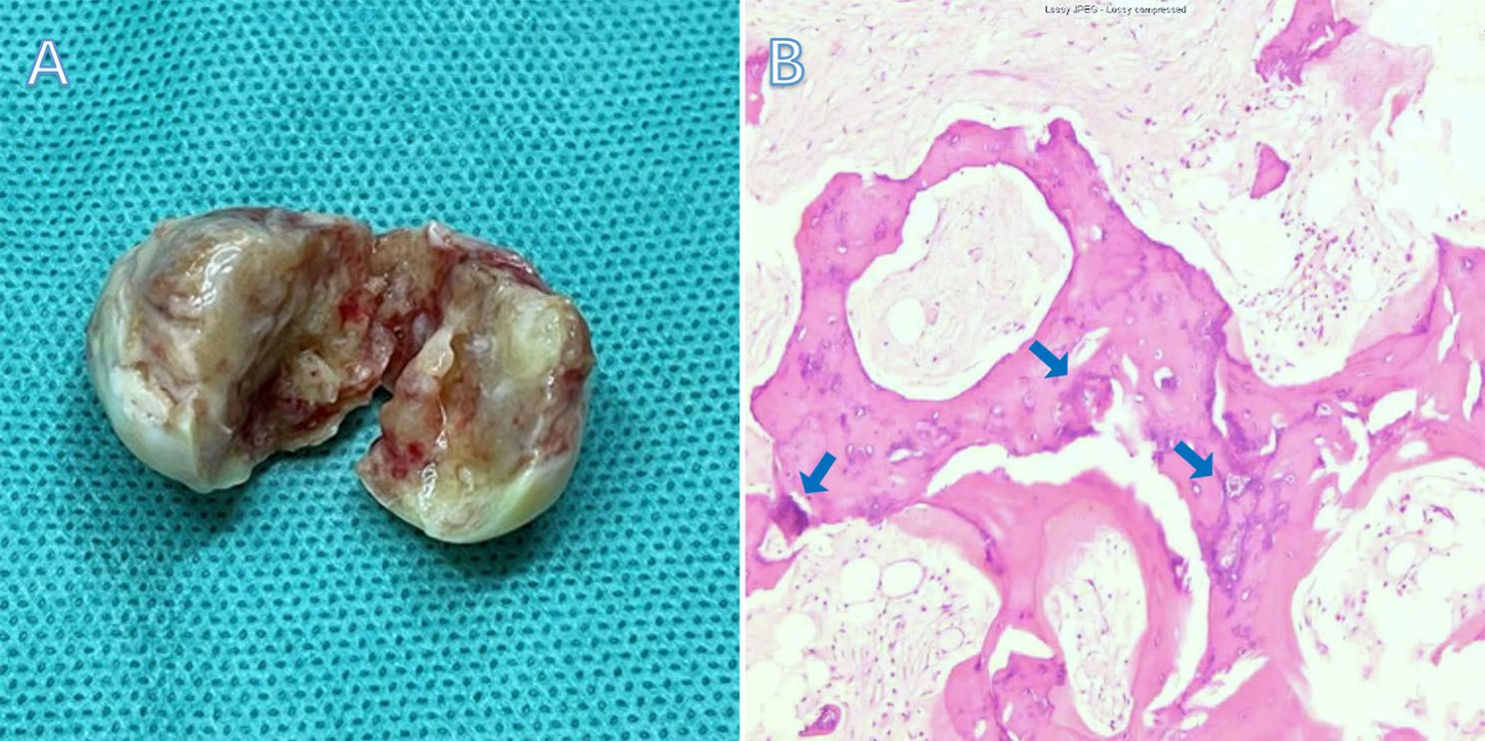
Macroscopic Appearance and pathological features of the mass. In the sagittal plane, the mass demonstrated a firm consistency and presented a gelatinous, semi-transparent cut surface (**A**). Left atrial solid myxoma with partial ossification (blue arrow indicating osteocyte formation) on the pathological section (**B**)

A 47-year-old woman, with no significant past medical history, was scheduled for surgery to remove a recurrent lipoma from her right upper arm. The physical examination and lab tests were normal. Preoperative transthoracic echocardiography (TTE) incidentally detected a mass in the left atrium, adjacent to the IAS. The mass appeared hyperechoic with significant posterior acoustic shadowing and demonstrated minimal mobility (Fig. 1A). Transesophageal echocardiography (TEE) revealed multiple short, rod-shaped, nodular-like hyperechoic lesions within the left atrial mass, which was associated with distal acoustic shadowing. The mass was without a stalk and was closely adherent from the upper aspect of the atrial septum to the apex of the left atrium. There was no blood flow signal within the lesion, and the mass showed a slight range of motion with the cardiac cycle (Fig. 1B-C). Contrast transthoracic echocardiography (cTTE) depicted the mass with extremely limited blood supply, with only a minimal amount of contrast agent infiltrating the lesion. Additionally, calcifications were present in both the peripheral and internal areas of the mass (Fig. 1D). The computed tomography angiography (CTA) revealed a heterogeneous enhanced mass in the left atrium, which was suspected to be a malignant tumor (Fig. 1E-F). The patient underwent surgical resection of the left atrial tumor. Intraoperatively, a grayish, irregular mass was identified within the left atrium. The mass was firm, encapsulated, with a broad base, and had a gritty texture upon palpation. The cut surface of the mass appeared partially transparent with a glial-like appearance (Fig. 2A). The pathology confirmed left atrial solid myxoma with partial ossification (Fig. 2B).

## Case 2

**Fig. 3:**
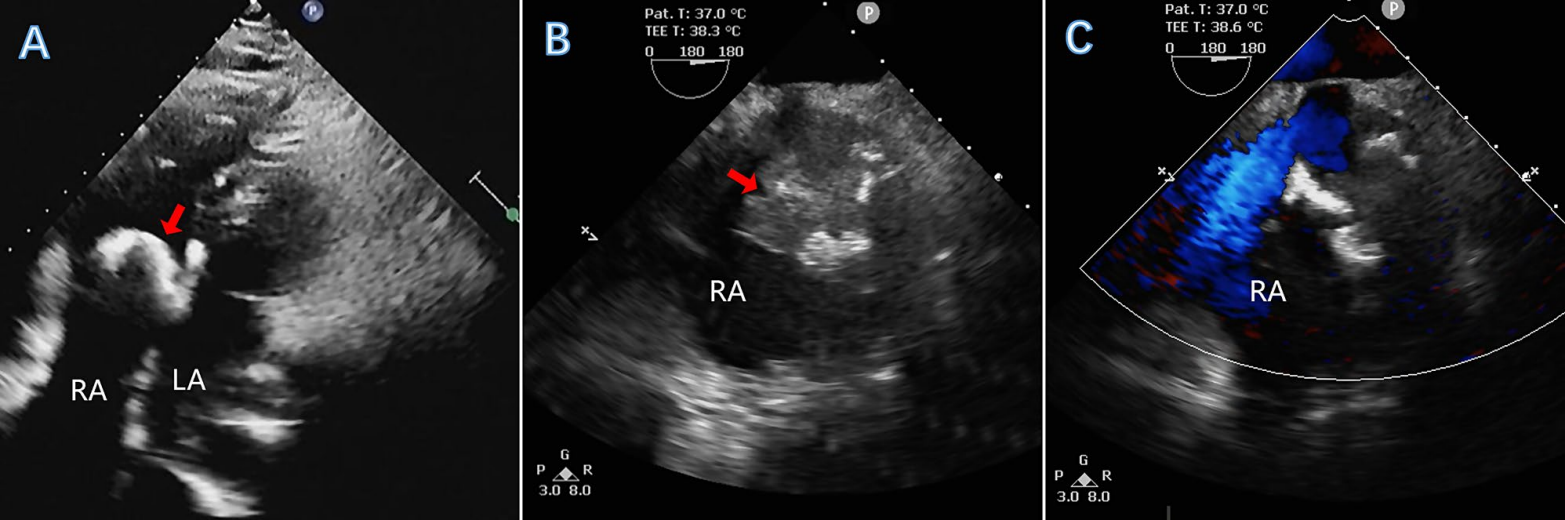
Multiparametric ultrasound images. On the four-chamber view obtained from both TTE and TEE at a 180° angle, a heterogeneous mass was identified in the right atrium, indicated by the red arrow (**A** and **B**). Within the mass, an arc-shaped hyperechoic focus was observed, accompanied by broad acoustic shadowing posterior to the mass. Color Doppler imaging revealed no significant blood flow within the mass (**C**)

**Fig. 4:**
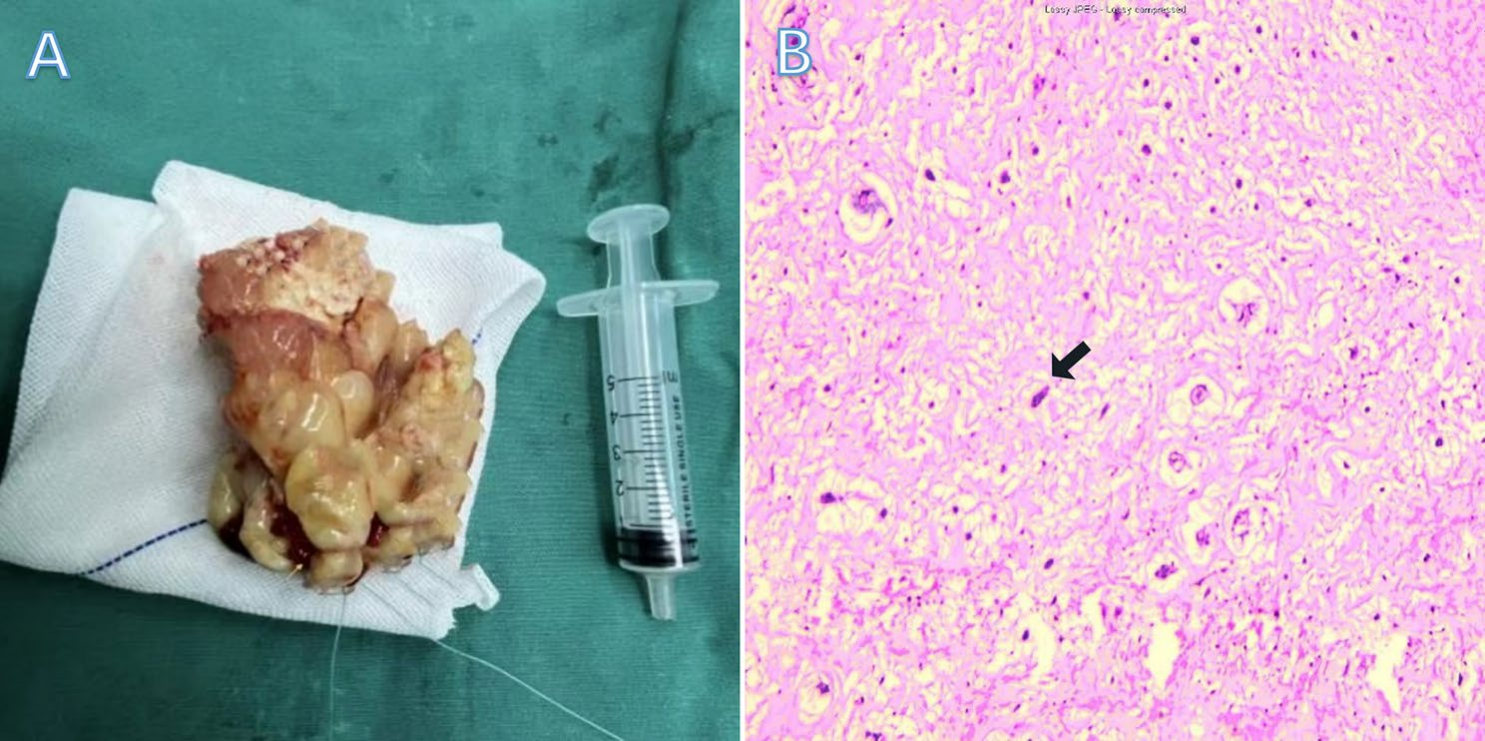
Macroscopic Appearance and pathological features of the mass. During surgery, a grayish-yellow, irregular mass was excised, characterized by multiple nodules and a grape-like surface morphology. The cut surface appeared solid and was gray-brown in color (**A**). The pathological section revealed left atrial myxoma with partial calcium deposition (black arrow) (**B**)

A 35-year-old male patient presented with proteinuria caused by chronic nephritis for more than 2 years and was scheduled for renal biopsy. The urinalysis showed proteins (3+), red blood cells (2+), and a microalbumin level of more than 0.15 g/L. The renal function revealed a uric acid level of 615.4 µmol/L and an antistreptolysin O titre of 355.0 IU/mL. TTE demonstrated a heterogeneous, echogenic mass in the right atrium, characterized by an irregular, large-sized morphology and a well-defined margin. The mass was pedunculated, with a stalk attaching it to the IAS, and exhibited substantial mobility throughout the cardiac cycle. During diastole, the mass obstructed the tricuspid valve orifice. Additionally, within the lesion, there was a band-shaped hyperechoic area accompanied by a broad acoustic shadow (Fig. 3A). TEE revealed that the right atrial mass exhibited extremely heterogeneous echogenicity, with multiple large arc-shaped hyperechoic foci within the lesion and distal acoustic shadowing posterior to the mass. The mass had a stalk situated between the right atrial appendage and the superior vena cava. The mass demonstrated significant mobility, prolapsing into the right ventricle during diastole and returning to the right atrium during systole. The tricuspid valve leaflets appeared thickened, obstructing normal opening, and severe regurgitation was evident during systole (Fig. 3B-C). The patient underwent resection of the right atrial mass followed by mechanical tricuspid valve replacement. During the operation, a grayish-yellow irregular mass was observed, with multiple nodules, a grape-like shape, and a solid gray-brown mass with significant calcification on the cut surface (Fig. 4A). The pathology confirmed right atrial solid myxoma with massive calcification (Fig. 4B).

## Discussion

CM is the most common benign tumor of the heart, often present as a yellowish, translucent, and gelatinous mass. Macroscopically, they can manifest as either solid or polypoid masses, typically attached to the IAS, or as papillary forms with an irregular surface. The papillary variants are particularly associated with an increased risk of systemic embolism due to their friable nature and potential for thrombus formation [4, 5]. Histologically, myxomas are heterogeneous, containing elements such as fibrosis, hemorrhage, ossification, calcification, and lipidation. Notably, calcification and ossification can occasionally be observed in solid myxomas, although they are not typical features. Calcification is present in approximately 20% of cases, while ossification is observed in about 8%[2, 6]. These occurrences, while relatively rare, pose diagnostic challenges on two-dimensional ultrasound due to the solid mass appearance and strong echogenicity accompanied by acoustic shadows.

The ossification of myxoma refers to the presence of osteoid tissue in the mass. Previous literature has reported that myxomas originate from primitive pluripotent mesenchymal stem cells at the fossa ovalis and endocardium of the heart. These cells can form multiple mesodermal lineages, such as osteoblasts, chondrocytes, adipocytes, myocytes, and vascular cells, to promote the ossification of myxomas [7]. In case 1, the tumor was attached to the atrial septum, extending to the apex of the left atrium, without a stalk. It exhibited little mobility and contained multiple, clustered, short rod-like hyperechoic, which were not consistent with the ultrasonographic feature of typical myxoma. Therefore, the possibility of teratoma was even considered after ultrasonography and intraoperative resection, and the mass was pathologically confirmed as solid myxoma with partial ossification.

The calcification of myxoma was caused by calcium deposition within the mass, and some authors called it “petrochemical atrial myxoma“ [8].possibly due to diseases with abnormal calcium and phosphorus metabolism, such as chronic nephritis and hyperparathyroidism [9].In case 2, the mass was attached to the right atrial wall near the superior vena cava, with a stalk and exhibiting large range of motion throughout the cardiac cycle. The tumor showed multiple and large arc-shaped hyperechogenic areas, accompanied by a broad, acoustic shadowing. Because the ultrasonographic findings were consistent with the characteristics of conventional myxoma and the patient had a history of chronic nephritis, the mass was diagnosed as myxoma, and the mass was later pathologically confirmed to be solid myxoma with massive calcification. There were significant differences between the echocardiography findings of calcification and ossification in our cases. However, whether this difference could be used as a differential point for atrial myxoma with ossification or calcification required further clinical studies.

TTE has some limitations in distinguishing calcification and ossification in CM from that observed in malignant tumors. For instance, it can be challenging to differentiate these features from calcifications seen in primary cardiac osteosarcomas, chondrosarcomas, and certain metastatic lesions [9, 10]. Other factors, such as the location, shape, composition, and vascularization of the masses, as well as the clinical and laboratory findings, should be considered for the differential diagnosis. CM typically originates from the atrial walls of the left or right atrium and the IAS, while malignant tumors can involve any cardiac chamber or structure. CM is benign and non-invasive, and does not affect the pericardium, while malignant tumors are aggressive and invasive, and can involve the pericardium. CM has a variable size, shape, and mobility, and may obstruct or prolapse across the atrioventricular valve, while malignant tumors are usually fixed and rarely interfere with valve function. CM has a variable growth rate and vascularization, and may present atypically with mitoses or pleomorphic cells, while malignant tumors have a rapid growth rate and high vascularization, and may show tumor-specific markers [11]. Therefore, While TTE may not provide sufficient diagnostic detail for atypical cases of CM, such as the right atrial myxoma with extensive calcification discussed in this paper, it is often necessary to supplement TTE with additional imaging modalities. These include angiography, cardiovascular magnetic resonance imaging (MRI), and CTA. Notably, TEE and cTTE offer distinct advantages by providing precise details on the tumor’s morphology, mobility, hemodynamics, blood supply, and attachment points. Furthermore, these methods mitigate the drawbacks associated with other diagnostic approaches, including invasiveness and radiation exposure. Complementing these techniques, MRI’s ability to depict the heterogeneous nature of myxomas, including areas of hemorrhage, fibrosis, and calcification, is particularly valuable. This non-invasive imaging tool enhances preoperative planning and risk assessment by offering a comprehensive view of the tumor’s composition and its potential impact on cardiac function.

## Conclusion

We present two rare cases of atrial myxoma with calcification or ossification and analyze their ultrasonographic features. We find that TEE and cTTE can provide valuable information for the diagnosis and management of such masses. However, the differentiation of calcification, ossification in myxoma, and malignant tumor calcification remains challenging. More studies are needed to understand the pathogenesis and imaging characteristics of these variants of myxoma, and we aim to deepen the understanding of our colleagues in ultrasonography regarding these types of myxomas and to offer a reference for the diagnostic assessment of similar cases.

### Electronic supplementary material

Below is the link to the electronic supplementary material.

Supplementary Material 1

## Data Availability

No datasets were generated or analysed during the current study.
